# Assessment of Histopathological Alterations and Oxidative Stress in the Liver and Kidney of Male Rats following Exposure to Aluminum Chloride

**DOI:** 10.1155/2024/3997463

**Published:** 2024-07-12

**Authors:** Anfal Kadhim, Ahlem Ben Slima, Ghusoon Alneamah, Mohamed Makni

**Affiliations:** ^1^ Environmental Sciences and Sustainable Development Laboratory LASED LR 18ES32 University of Sfax, Sfax, Tunisia; ^2^ Department of Food Technology High Institute of Biotechnology of Sfax University of Sfax, Sfax, Tunisia; ^3^ Department of Pathology College of Veterinary Medicine University AL-Qasim Green, Al Qasim, Iraq

## Abstract

The study aims to investigate the residual and histopathological effects of chronic aluminum chloride (AlCl_3_) toxicity in the kidney and liver of male rats. After 30-, 60-, and 90-day exposure period, analyses were conducted to assess the toxicity in the kidney and liver. The results showed that the concentration of AlCl_3_ in the kidney and liver increased significantly in 30-, 60-, and 90-day periods. The effects of oxidative stress on the kidneys and liver were dose- and time-dependent. Levels of malondialdehyde (MDA) significantly increased when exposed to AlCl_3_ groups. Conversely, the activity of antioxidant parameters, including reduced glutathione (GSH), catalase (CAT), and superoxide dismutase (SOD), significantly decreased in the AlCl_3_ exposed groups, indicating compromised oxidant mechanisms. Both the kidney and liver exhibited severe tissue damage, including necrosis, fibrosis, and inflammatory cell infiltration, in rats exposed to AlCl_3_. Kidney sections showed hyperplasia of the epithelial cells lining the renal tubules, resembling finger-like structures. Liver sections displayed severe lobular hyperplasia and an increase in mitotic figures. Our study suggests that AlCl_3_ has a detrimental impact on these vital organs and emphasizes the importance of monitoring and mitigating aluminum exposure, particularly where it is present in high concentration.

## 1. Introduction

Aluminum (Al) is one of the most abundant elements on Earth and is commonly found in various natural and industrial sources. While aluminum is relatively inert in its elemental form, it can become toxic when present in soluble or bioavailable forms. Human exposure to aluminum compounds has increased over the years, primarily due to its presence in a wide range of products, such as food additives, drinking water, antacids, and cookware. It is the third most abundant metal in the Earth's crust, present in large quantities in soil and water [1].

Aluminum compounds are ingested through the consumption of food and beverages; they are commonly used as food additives to improve the texture and appearance of processed foods. These compounds can be found in baking powder, self-rising flour, cake mixes, and some processed cheeses. Aluminum can enter the food supply through water sources, as it is sometimes used in water treatment processes [2, 3]. This can lead to aluminum contamination in drinking water and, subsequently, in food and beverages prepared with that water. In some regions, naturally occurring aluminum in soil and rock can leach into groundwater and surface water, leading to elevated aluminum levels in drinking water [4]. Aluminum-based coagulants, such as aluminum sulfate, are used in water treatment to clarify water by removing impurities. While this is an essential process for water purification, residual aluminum levels in treated water can vary and potentially pose health concerns when consumed over a long period.

Moreover, some vaccines contain aluminum compounds as adjuvants to enhance the body's immune response to the vaccine. These include certain vaccines used in childhood immunization programs. Aluminum hydroxide is a common ingredient in antacids and other over-the-counter medications used to treat heartburn, indigestion, and acid reflux. Prolonged use of such medications can lead to aluminum intake [5]. Among children, authors found that vaccine-associated aluminum exposure is in favor of those children would be more than two and half times more likely to develop persistent asthma than those without this exposure [6].

It is important to note that the overall impact of aluminum exposure from these sources can vary significantly. While acute exposure to aluminum is generally not a cause for concern, chronic exposure, especially at high levels, may be associated with health risks, including hepatotoxicity, neurotoxicity, and potential links to conditions such as Alzheimer's disease [3, 7, 8].

Regulatory agencies, such as the World Health Organization and the U.S. Environmental Protection Agency, provide guidelines and regulations to address aluminum levels in drinking water and food additives, aiming to minimize potential health risks associated with aluminum exposure. The typical concentration of airborne aluminum ranges from 0.0005 *μ*g/m^3^ in Antarctica to over 1 *μ*g/m³ in industrialized regions [4]. In natural waters, dissolved aluminum levels (at pH 7) typically range from 0.001 to 0.05 mg/L but can elevate to 0.5–1 mg/L in acidic or organically rich waters. With an average adult dietary intake of 5 mg/day of aluminum from food and a drinking water aluminum concentration of 0.1 mg/L, drinking water accounts for approximately 4% of total oral aluminum exposure. Generally, the contribution of air to overall aluminum exposure is minimal [4].

Globally, data on aluminum intake vary significantly due to multiple factors. For instance, adult dietary aluminum intake ranges from 7.1–8.2 mg/day in the USA to 4.5 mg/day in Japan. Children's intake is reported at 0.8 mg/day in Germany, while in the United Kingdom and in the USA, it varies from 0.03 to 0.7 mg/day. In Germany, aluminum levels in public water supplies average 0.01 mg/l in the western region, but 2.7% of supplies in the eastern region exceed 0.2 mg/l [4]. Cosmetics are deemed safe with aluminum concentrations of 2.65% in toothpaste and 0.77% in lipstick [9], and vaccines licensed in the USA are considered safe when containing aluminum ranging from 0.85–0.125 mg per dose [10].

In the small intestine, aluminum chloride is rapidly converted to insoluble poorly absorbed basic aluminum salts, consisting of a mixture of hydrated aluminum oxide, oxyaluminum hydroxide, various basic aluminum carbonates, and aluminum soaps [11]. Accumulation of aluminum in the brain has been associated with neurotoxicity and is of particular concern regarding its potential role in neurodegenerative diseases such as Alzheimer's disease. While the exact mechanisms are not fully understood, aluminum can induce oxidative stress and may contribute to the development or progression of neurological disorders [7, 12]. Chronic exposure to aluminum can have adverse effects on bone health as aluminum interferes with bone mineralization and can lead to bone demineralization, potentially contributing to conditions such as osteoporosis and osteomalacia [13, 14]. The kidneys play a critical role in filtering and excreting aluminum from the body. Chronic exposure to aluminum can lead to renal dysfunction and damage, impairing the kidney's ability to filter waste products effectively [15, 16]. Inhalation of aluminum dust or ingestion of large amounts of aluminum compounds can lead to respiratory and gastrointestinal issues. However, these effects typically result from acute exposure to high levels, such as in occupational settings [4, 17].

It is important to note that the potential health effects of aluminum exposure are a subject of ongoing research and debate. While there is evidence of aluminum's toxic potential, it is essential to consider the specific source, duration, and concentration of exposure, as well as individual susceptibility.

The objective of this study is to determine the residual, histopathological, and oxidative effects of chronic aluminum chloride toxicity on the kidney and liver in rats.

## 2. Materials and Methods

### 2.1. Aluminum Chloride

Aluminum chloride hexahydrate (AlCl_3_ 6 H_2_O) powder was obtained from Sigma-Aldrich Chemical Co. (St. Louis, USA) (A0718), and all other chemicals used were of analytical grade. Aluminum ion treatment concentration was prepared by dissolving (2.7 g) aluminum chloride in 40 ml distilled water. Dissolved AlCl_3_ is administered orally at a dose of 100 and 200 mg/kg bw. for 30, 60, and 90 days. The doses of AlCl_3_ were selected according to the research study conducted by Kumar et al. [18].

### 2.2. Experimental Animals

A total of 90 male Wistar rats, aged between 4 and 9 weeks and weighing between 200 and 300 g, were obtained from the National Center for Drug Control and Research in Baghdad (Iraq). They were housed at animal house of the Veterinary Medicine College in Baghdad (Iraq) for 2 weeks before starting the experiment by rearing in separated, cleaned, and disinfected cages, and they were fed on commercial assorted pellets and clean water for drinking. All researchers are obligated to ensure the well-being of animals in their care, in strict adherence to the highest standards, and in accordance with Baghdad University laws, regulatory guidelines, and humane principles. In our study, the local animal welfare committee approved the current protocol.

Subsequently, the rats were randomly divided into three groups, with 30 per group. The first group of rats (GI: control) served as the controls and received ad libitum distilled water and standard diet. The second group (GII: 100 mg/kg) had received orally the dose of 100 mg/kg bw. of AlCl_3_ for 30, 60, and 90 days. Also, the third group (GIII: 200 mg/kg) had received orally the dose of 200 mg/kg bw. of AlCl_3_ for 30, 60, and 90 days. Body weight and food intake were recorded every 6-7 days (Supplementary Table 1), and at the end of the experimental period (30, 60, or 90 days), the animals in different groups were sacrificed by cervical decapitation to avoid stress conditions. The liver and the kidney were quickly excised, rinsed in ice-cold physiological saline, weighed, and then divided into parts: one for homogenization in the appropriate buffer at 10% (w/v) as indicated in the procedure's measurement of each parameter. The supernatant aliquots were stored at −80°C and used for biochemical assays, one was kept in formalin for histopathological analysis, and the last part was kept as such and put together at −80°C until needed.

### 2.3. Determination of Aluminum in the Tissue Samples by Flameless Atomic Absorption Spectrophotometer

The initial standard solution for the desired element was 1000 *μ*g/ml in 2% HNO_3_. The trace element (aluminum) was determined using the flameless atomic absorption spectrophotometer method using the graphite furnace (GFAAS) technology. Four standard solutions (1000 *μ*g/ml, 100 *μ*g/ml, 10 *μ*g/ml, and 1 *μ*g/ml) were obtained by diluting the initial standard stock solution. A sequence of concentrations starting at the highest level and descending to the amounts necessary for the calibration curve's performance must be prepared. The sample preparation is conducted by digestion of the animal tissue method [19]. One gram of liver and kidney tissues was weighed and put into a 100 ml conical flask. Then, five milliliters of concentrated nitric acid (HNO_3_ 70%) and one milliliter of perchloric acid (HClO_4_) were added, and the sample was allowed to sit at room temperature for one hour. Samples were placed on a heated plate at 100°C until violet vapors started to form, at which point, the temperature was increased to 150–200°C until white fumes started to appear. The samples were filtered using paper (0.45), and the volume was adjusted with distilled water that had been acidified with 1% HNO_3_ up to the point when the remaining solution, which was pale yellow, indicated that the digestion process had been completed (25 ml). Results were read using the graphite furnace technique of atomic absorption spectrophotometer.

### 2.4. Determination of Oxidant and Antioxidant Parameters by ELISA (Enzyme-Linked Immunosorbent Assay)

The double antibody sandwich technique of the ELISA was performed according to the My BioSource manufacturer's recommendation kits.

The preparation of tissue homogenates varies depending upon the tissue type. Tissues were collected and weighed before homogenization, minced to small pieces, and homogenized with a PBS (usually 10 mg tissue to 100 *μ*l PBS.). The homogenate is then centrifuged at 1000 × g (3000 rpm) for 20 minutes. The supernatant was collected carefully, and immediately samples were stored at −80°C until use.

The ELISA analytical biochemical technique of the (MBS738685) kit for MDA, the (MBS701908) kit for catalase (CAT), the (MBS036924) kit for superoxide dismutase (SOD), and the (MBS2700076) kit for glutathione (GSH) is based on the enzyme-linked immunosorbent assay, which is to measure antigens by fixing antibodies associated with enzymes on them. The principle of this technique is based on the visualization of an antigen-antibody reaction using a colorimetric enzymatic reaction. The enzyme, previously coupled to the antibody, catalyzes a colored chemical reaction which transforms its substrate into a compound. After removing any unbound substances, a biotin-conjugated specific antibody is added to the wells. After washing, avidin-conjugated horseradish peroxidase (HRP) is added to the wells. Following a wash to remove any unbound avidin-enzyme reagent, a substrate solution is added to the wells and the color develops in proportion to the amount of molecules bound in the initial step. The color development is stopped, and the intensity of the color is measured by spectrophotometer (Cecil, France) at 450 nm in a microplate reader (Biotech, USA).

### 2.5. Histopathological Study

Liver and kidney samples, intended for histological examination, were processed overnight for dehydration, clearing, and impregnation using an automatic tissue processor (Sakura, Japan). The specimens were embedded in paraffin blocks using an embedding station (Sakura, Japan) and serial sections of 5 *µ*m thickness were cut using a microtome (ModelRM2245, Leica Biosystems, Wetzlar, Germany) and stained by hematoxylin/eosin as described by Bancroft and Gamble [16]. Once dried, the sections were observed under a light microscope (Imager A2, Zeiss, Gottingen, Germany) at a magnification of 400× and photographed using a digital camera.

### 2.6. Statistical Analysis

The Statistical Analysis System program was used to detect the effect of different factors in study parameters (comparing the experimental group with the control group, each one containing 30 animals) [20]. Least significant difference (LSD) test using analysis of variance (ANOVA). Results were presented as mean ± the standard deviation. The level of significance used in the analysis was set at *p* ≤ 0.05, indicating a statistically significant difference between groups.

## 3. Results

### 3.1. Residual of AlCl_3_ in the Liver and Kidney

The determination by flameless atomic absorption of aluminum in the kidney and liver for toxicological monitoring of rats treated with the two different concentrations (100 and 200 mg/kg) is illustrated in Tables 1 and 2, respectively. “An accumulation of aluminum was observed in the kidneys of treated rats, which significantly increased (*p* ≤ 0.05) with the duration of exposure at a dose of 100 mg/kg. The aluminum concentration ranged from 1.88 ± 0.30 ppm during the first 30 days to 8.08 ± 0.07 ppm after 90 days of exposure. In addition, the aluminum levels increased with higher concentrations of aluminum exposure across all study periods (30, 60, and 90 days) compared to the control group (0.034 ± 0.01 ppm, 0.037 ± 0.01 ppm, and 0.033 ± 0.01 ppm, respectively) (Table 1).”

The exposure at 200 mg/kg is more harmful and significant increase (*p* ≤ 0.05), ranging from 2.91 ± 0.01 ppm during the first 30 days to 17.82 ± 0.11 ppm after 90 days of exposure and with increasing concentration of aluminum exposure during all periods studied 30, 60, and 90 days compared to control (Table 1).

Significantly elevated aluminum levels are measured in the livers of treated rats depending on the increasing concentration studied (0.036 ± 0.00 ppm to 2.86 ± 0.02 ppm for 30 days; 0.033 ± 0.00 to 4.71 ± 0.05 ppm for 60 days, and 0.030 ± 0.00 to 12.84 ± 0.74 ppm for 90 days) (Table 2).

This observation is consistent with the previous research study indicating that aluminum, when ingested, can accumulate in various tissues, especially the kidney, which plays a crucial role in filtering and excreting aluminum from the body. Such studies indicate that the oral bioavailability of aluminum from dietary sources is projected to be somewhat lower or similar compared to previous research studies focused on aluminum exposure through water, in both human and rat studies [2]. The prolonged exposure in this study likely allowed for the gradual accumulation of aluminum in these organs.

### 3.2. Oxidative Stress Aluminum Chloride in the Kidney and Liver

**Figure 1:**
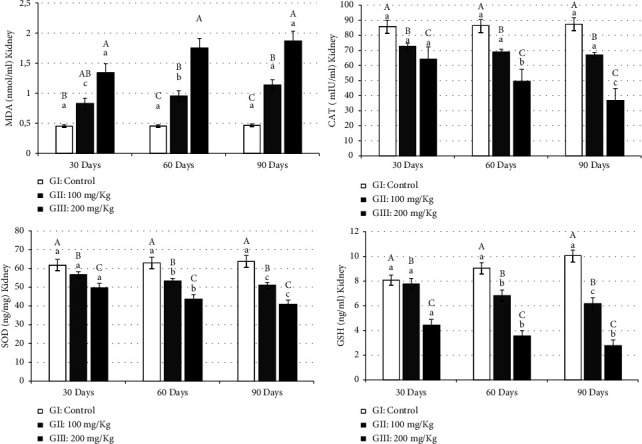
Oxidant parameters in the kidney of rats treated by AlCl_3_. Results are expressed as mean ± standard deviation. Means with the same letter are not significantly different. Means with different big letters in the same column and small letters in the same row are significantly different (^*∗*^*p* ≤ 0.05).

**Figure 2:**
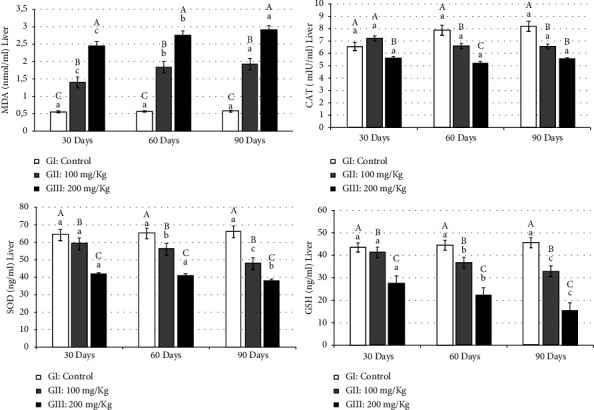
Oxidant parameters in the liver of rats treated by AlCl_3_. Results are expressed as mean ± standard deviation. Means with the same letter are not significantly different. Means with different big letters in the same column and small letters in the same row are significantly different (^*∗*^*P* ≤ 0.05).

Lipid peroxidation, confirmed by high levels of MDA, serves as a marker of oxidative damage. Aluminum-exposed rats exhibited a significant increase (*p* ≤ 0.01) in MDA levels in both the kidneys (Figure 1) and liver (Figure 2). This increase in MDA levels correlated with the duration of the treatment period, indicating a progressive rise in oxidative damage over time. The significant increase in MDA levels also corresponded to higher aluminum concentrations across the three periods (30, 60, and 90 days) in the kidney (GII: 0.83 ± 0.04, 0.95 ± 0.01, and 1.14 ± 0.04 and GIII: 1.33 ± 0.02, 1.75 ± 0.03, and 1.87 ± 0.02) as well as in the liver (GII: 1.39 ± 0.05, 1.83 ± 0.03, and 1.92 ± 0.02 and GIII: 2.44 ± 0.05, 2.75 ± 0.02, and 2.90 ± 0.06) than in the control group in the kidney (0.45 ± 0.04, 0.46 ± 0.012, and 0.47 ± 0.04) and liver (0.54 ± 0.02, 0.55 ± 0.04, and 0.56 ± 0.01), respectively.

The reduction in the kidney CAT activity induced by aluminum compared to the control group (GI: 85.56 ± 0.04, 86.23 ± 0.02, and 87.25 ± 1.91; GII: 72.83 ± 0.55, 69.03 ± 2.23, and 66.73 ± 5.05), especially in rats treated with 200 mg/kg of aluminum (GIII: 64.24 ± 3.24, 49.53 ± 0.35, and 36.83 ± 1.29), reflects a decrease in the capacity of these organs' mitochondria and microsomes to eliminate H_2_O_2_. Similar results were observed in the liver of aluminum-treated rats (GII: 7.20 ± 0.57, 6.59 ± 0.14, and 6.56 ± 0.12 and GIII: 5.61 ± 0.07, 5.21 ± 0.08, and 5.53 ± 0.35) (Figure 1).

SOD is considered the first line of defense against the deleterious effects of free radicals in cells, catalyzing the dismutation of superoxide radicals to hydrogen peroxide and molecular oxygen. Kidney SOD activity declined significantly (*p* ≤ 0.01) in aluminum-treated animals (GII: 56.53 ± 1.51 to 50.76 ± 0.15 and GIII: 49.33 ± 0.81 to 40.36 ± 0.06) compared to control animals (GI: 61.6 ± 0.69, 62.6 ± 1.65, and 63.5 ± 2.21) (Figure 1). A reduction in SOD levels was also observed in the liver of rats treated with two concentrations of aluminum (GII: 59.13 ± 0.99, 56.06 ± 0.76, and 37.86 ± 0.74 and GIII: 41.6 ± 0.70, 40.76 ± 0.12, and 37.86 ± 2.00) (Figure 2).

Aluminum treatment led to a significant decrease (*p* ≤ 0.01) in GSH in both the kidney (GII: 7.75 ± 0.03, 6.83 ± 0.05, and 6.19 ± 0.03 and GIII: 4.42 ± 0.41, 3.53 ± 0.44, and 2.78 ± 0.44) (Figure 1) and the liver (GII: 41.16 ± 1.15, 36.56 ± 1.22, and 32.86 ± 2.11 and GIII: 27.3 ± 0.53, 21.96 ± 2.98, and 15.23 ± 0.35) (Figure 2) compared to the control group in the kidney (GI: 8.06 ± 0.70, 9.03 ± 0.42, and 10.02 ± 0.93) and the liver (GI: 44.33 ± 2.08, 44.36 ± 1.33, and 45.41 ± 1.21), with decreases observed over the treatment period (Figure 2). GSH is secreted from hepatocytes into the bile canalicular spaces. In the kidneys, GSH is degraded by membranous *γ*-GT and other peptidases. This relationship explains the positive correlation of GSH levels found in the kidneys and liver of rats exposed to aluminum.

These findings suggest that chronic AlCl_3_ exposure induces oxidative stress in the kidney and liver, compromising antioxidant defense mechanisms and leading to cellular damage, as confirmed by our findings. Our results are consistent with those reported by Bondy [12] and Savory [16]. They found that even in the group receiving the lowest aluminum content, the effect of chronic AlCl_3_ exposure on lipid peroxidation levels was pronounced, with a highly significant difference.

### 3.3. Histopathological Effects of Aluminum Chloride in the Kidney and Liver

**Figure 3:**
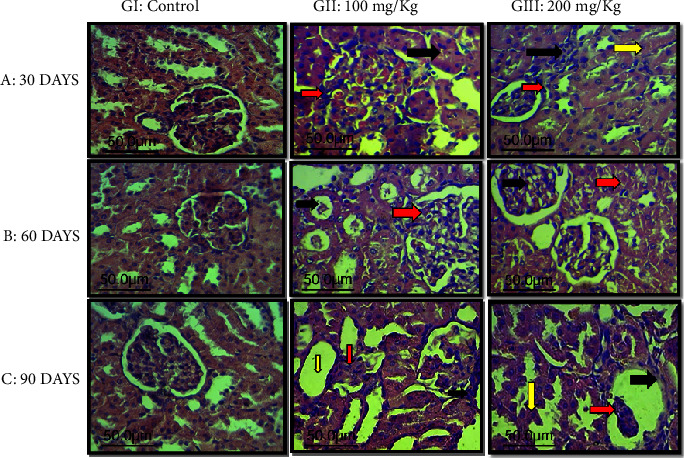
Histopathological sections of rat kidney elapsed time after AlCl_3_ treatment with GI: control; GII: 100 mg/kg bw of AlCl_3_; GIII: 200 mg/kg bw of AlCl_3_; (A: 30 days; B: 60 days; C: 90 days) (H&E stain, 400x). (A): 30 days: GI: control group. GII: obliteration of glomerular tufts (red arrow) with cloudy swelling of the proximal convoluted renal tubules (black arrow). GIII: mild thickening of Bowman's capsule (red arrow) with degeneration changes in the renal tubules (yellow arrow) and inflammatory cell infiltration (black arrow). (B) 60 days: GI: control group. GII: vacuolation and congestion of the glomerular tuft (red arrow), with deposition of the protein material inside the tubular lumen (black arrow). GIII: necrosis in the proximal convoluted tubules (red arrow), with vacuolation and congestion of the glomerular tuft (black arrow). (C) 90 days: GI: control group. GII: marked atrophy of glomerular tuft (red arrow) with thickening of Bowman's capsule (black arrow) and cystic dilation in the renal tubules (yellow arrow). GIII: severe collapse of the glomerular tuft (red arrow) with thickening of Bowman's capsules (black arrow) with hyperplasia of the epithelial cells lining the renal tubules (appears as finger-like structures) (yellow arrow).

**Figure 4:**
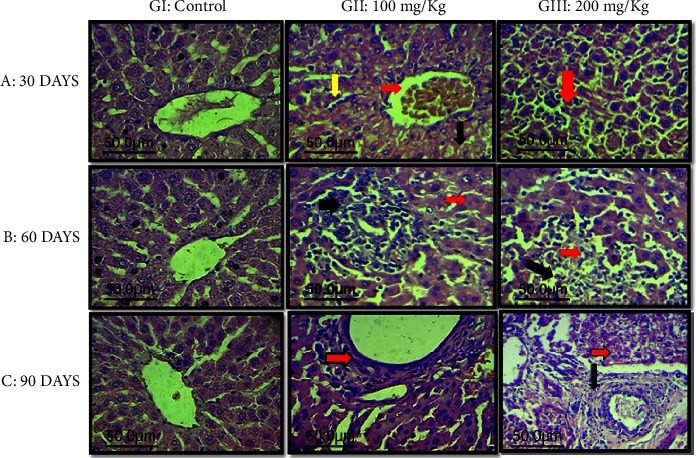
Histopathological sections of rat liver elapsed time after AlCl_3_ treatment with GI: control; GII: 100 mg/kg bw of AlCl_3_; GIII: 200 mg/kg bw of AlCl_3_ (A: 30 days; B: 60 days; C: 90 days) (H&E stain, 400x). (A) 30 days: GI: control group. GII: congestion of the central vein (red arrow), with mild centrilobular necrosis and inflammatory cells infiltration (black arrow) and proliferation of Kupffer cells (yellow arrow). GIII: dilation of sinusoid with inflammatory cells infiltration (red arrow). (B) 60 days: GI: control group. GII: single cell necrosis (red arrow) with extensive periductal mononuclear cells aggregation (black arrow). GIII: extensive area of necrosis (red arrow) and apoptosis (black arrow). (C) 90 days: GI: control group. GII: marked thickening in the central vein due to possible fibrosis and inflammatory cells infiltration (red arrow). GIII: severe aggressive hepatic necrosis (red arrow) with severe probably periportal fibrosis (black arrow).

Chronic exposure to aluminum chloride can lead to histopathological changes in the kidney (Figure 3) and liver (Figure 4) architectures, which are observable. These changes include hepatic inflammation, potential hepatic fibrosis, altered hepatocyte architecture, the formation of granulomas or fatty changes, tubular damage in the kidney, interstitial inflammation, glomerular alterations, and possibly fibrosis in renal tissues.

In our study, both the kidney and liver exhibited severe tissue damage in rats exposed to both 100 mg/kg and 200 mg/kg of AlCl_3_ over 30 (A), 60 (B), and 90 days (C).

Kidney sections showed hyperplasia of the epithelial cells lining the renal tubules, which resembled finger-like structures. Liver sections displayed severe lobular hyperplasia and an increase in mitotic figures. Our results are consistent with previous studies [3, 14].

Hyperplasia of the epithelial cells lining the renal tubules refers to an increase in the number of cells in the kidney, leading to enlargement of the affected area. This description likely refers to the architectural appearance of hyperplastic tubular cells. Normally, these cells are somewhat uniform, but with hyperplasia, they can proliferate excessively and irregularly, potentially forming projections or expansions that resemble fingers.

Liver hyperplasia refers to an increase in the number of liver cells, leading to the enlargement of the organ. It is characterized by the diffuse transformation of the liver architecture into small nodules. This condition occurred due to chronic exposure to AlCl_3_, which affects the liver's ability to regenerate and respond to injury or increased functional demands.

We found that animals in the control groups (GI) exhibited normal histological structure (Figures 3 and 4). In contrast, animals in GII, treated with 100 mg/kg bw AlCl_3_ for 30, 60, and especially 90 days, showed marked atrophy of the glomerular tuft with thickening of Bowman's capsule and cystic dilation in the renal tubules (Figure 3). In the liver, there was marked thickening in the central vein, likely due to fibrosis and infiltration of inflammatory cells (Figure 4).

Animals in GIII, treated with 200 mg/kg bw AlCl3 for 30, 60, and especially 90 days, exhibited severe collapse of the glomerular tuft with thickening of Bowman's capsule and hyperplasia of the epithelial cells lining the renal tubules, forming finger-like structures (Figure 3). Liver sections showed severe lobular hyperplasia (Figure 4).

## 4. Discussion

The findings concerning the accumulation of AlCl_3_ in the kidney and liver and its potential role in aluminum poisoning have garnered significant interest in recent years. Aluminum is known to persist in the environment and can readily accumulate in various body tissues, posing potential harm to target organs such as the liver, kidneys, spleen, heart, and brain. This phenomenon has been supported by several studies. Many studies have highlighted the persistence of AlCl_3_ in the body and its tendency to accumulate in critical organs [21]. Other researchers have emphasized aluminum's potential to accumulate in various tissues, contributing to adverse effects on the body [22, 23]. Yu et al. observed that within the initial eight weeks of aluminum exposure, the liver and kidneys primarily accumulate aluminum [24]. This finding underscores how quickly aluminum can be sequestered by these organs.

The kidney tends to accumulate higher levels of aluminum than the liver, depending on the dose and exposure duration, which aligns with the fact that the kidney plays a pivotal role in eliminating aluminum from the body. Approximately 95% of aluminum elimination occurs through urine, while the biliary pathway contributes only about 2% to total aluminum excretion [25]. This finding is consistent with the concept that kidney disease can impede aluminum removal, potentially leading to more pronounced aluminum accumulation in the kidneys than in the liver [26–28].

These findings collectively underscore the significance of understanding the dynamics of aluminum accumulation in vital organs, particularly the kidney and liver, and its potential health implications.

The vulnerability of vital organs, such as the liver and kidneys, to metal poisoning is well documented. Aluminum has shown to induce oxidative stress in these tissues, leading to adverse effects on cellular and tissue health. Oxidative stress occurs when there is an imbalance between the production of reactive oxygen species (ROS) and the body's ability to neutralize them with antioxidants. In the case of aluminum exposure, it has been observed that aluminum can reduce the activity of the glutathione-synthase enzyme, resulting in decreased levels of glutathione (GSH), an essential antioxidant in the body. Consequently, the antioxidant defense mechanisms in tissues become compromised. Other studies provide valuable insights into the mechanisms by which aluminum induces oxidative stress in tissues [29, 30]. Aluminum may inhibit NADPH-generating enzymes such as glucose 6-phosphate dehydrogenase, which are crucial for GSH regeneration [31].

Moreover, our findings showed an increase in malondialdehyde (MDA) levels and a decrease in the activities and levels of antioxidant molecules, including superoxide dismutase (SOD), catalase (CAT), and glutathione (GSH), both in the kidney and liver. These findings suggest a clear link between aluminum exposure, oxidative stress, and the body's antioxidant defense mechanisms. The rise in MDA levels in the kidney and liver indicates lipid peroxidation, which is a hallmark of oxidative stress and confirms that these tissues are experiencing oxidative damage due to chronic aluminum exposure [32].

The reduction in the levels and activities of antioxidant molecules such as SOD, CAT, and GSH is concerning. These molecules play a vital role in neutralizing reactive oxygen species (ROS) and protecting cells and tissues from oxidative damage. A decrease in their levels suggests that the body's natural defense mechanisms against oxidative stress are compromised.

The observed effects are dose- and time-dependent, indicating that the extent of oxidative stress and impairment of antioxidant defenses increase with higher doses and longer exposure to aluminum. This finding underscores the importance of considering both the concentration of aluminum and the duration of exposure when assessing its potential health effects.

Studies have demonstrated that aluminum (Al) salts accelerate the peroxidation of membrane lipids induced by Fe (II) salts, suggesting that Al+3 interacts directly with cell membranes [33]. Al+3 ions subtly alter the membrane structure, enhancing iron's oxidative activity. Aluminum has been found to increase the rate of lipid peroxidation in various tissues such as the liver, kidney, testis, and brain of different animals [34–36].

Another study found that mice receiving aluminum treatment for three months showed higher levels of MDA, while GSH levels decreased [37]. Yi-Fei et al. reported that high doses of Al(NO_3_)_3_ induced an increase in lipid peroxidation rates. Even after brief exposure to Al(NO_3_)_3_, mice exhibited signs of oxidative stress, including elevated lipid peroxidation (LPO), decreased GSH levels, and inhibited SOD and CAT activity in the liver and kidney [38]. In addition, the deficiency of antioxidant enzymes contributes to increased lipid peroxidation, which in turn damages cells.

Histological examination of the liver and kidney in the context of aluminum toxicity often reveals specific structural changes indicative of damage and dysfunction. The findings of our study, which include histological lesions in the liver and kidney, are consistent with recent research study. These results underscore the potential harm that chronic exposure to AlCl_3_ can cause to vital organs [39].

The histological lesions observed in the liver, such as diffuse hepatocellular regeneration, central vein congestion, and sinusoidal congestion in the centrilobular zone, indicate liver damage. Aluminum exposure leading to centrilobular necrosis, characterized by the death of hepatocytes, particularly in the centrilobular region, is a mark of liver damage due to toxic insult [40]. Chronic aluminum exposure can also result in fatty infiltration of the liver, marked by the accumulation of fat droplets within hepatocytes, impairing liver function [41]. In addition, chronic aluminum exposure may lead to possible liver fibrosis, the accumulation of excess connective tissues in the liver, which disrupts its architecture and impairs function [42]. Such changes can disrupt normal liver function and may be associated with the accumulation of aluminum in the liver.

The liver sustains damage in the form of diffuse hepatocellular regeneration, central vein congestion, and sinusoidal congestion in the centrilobular zone. These structural changes indicate liver dysfunction, disrupted blood flow, and inflammation in response to aluminum exposure.

In the kidney, observed congestion in the capillary tuft of the glomeruli, diffuse vacuolar degeneration in epithelial cells, and focal cystic changes suggest kidney damage. Aluminum exposure can lead to glomerular changes, such as glomerulosclerosis, characterized by thickening and scarring of glomerular basement membranes [43]. Chronic aluminum exposure may also result in interstitial fibrosis in the kidney, the accumulation of fibrous tissues in the spaces between tubules and blood vessels [44]. Aluminum can cause injury to tubular epithelial cells in the kidney, marked by cellular swelling, degeneration, and loss of brush border [45]. These histological alterations are consistent with the potential impact of chronic aluminum exposure on renal tissues.

The histological changes observed in our study, supported by several other studies, reflect the diverse effects of aluminum exposure on the liver and kidney. These findings provide scientific evidence for our observations and underscore the importance of further research to better understand the mechanisms and potential health consequences of aluminum toxicity in tissues.

## 5. Conclusions

Chronic aluminum toxicity exerts significant and detrimental effects on both the liver and kidney, involving oxidative stress and structural damage to these vital organs. The combination of these factors highlights the potential health risks associated with prolonged exposure to aluminum compounds.

These findings underscore the multifaceted and interconnected consequences of aluminum toxicity. Oxidative stress and structural damage in the liver and kidney are interlinked processes, where oxidative stress can exacerbate tissue damage and compromise organ function. The adverse effects observed in both organs emphasize the critical need for monitoring and regulating aluminum exposure, especially in environments where it is prevalent, such as certain occupational settings and sources of environmental contamination.

## Figures and Tables

**Table 1:** Residue of aluminum chloride in the kidney for chronic toxicity in experimental groups.

Groups	Mean ± SE			LSD value
	30 days	60 days	90 days	
GI: control	0.034 ± 0.01^Ca^	0.037 ± 0.01^Ba^	0.033 ± 0.01^Ca^	0.011^NS^
GII: 100 mg/kg	1.88 ± 0.30^Bc^	4.60 ± 0.37^Ab^	8.08 ± 0.07^Ba^	0.965^*∗*^
GII: 200 mg/kg	2.91 ± 0.01^Ac^	5.37 ± 0.21^Ab^	17.82 ± 0.11^Aa^	0.481^*∗*^
LSD value	0.60^*∗*^	0.853^*∗*^	0.274^*∗*^	—

Results are expressed as mean (ppm) ± standard deviation. Means with the same letter are not significantly different. Means with different big letters in the same column and small letters in the same row are significantly different (^*∗*^*p* ≤ 0.05).

**Table 2:** Residue of aluminum chloride in the liver for chronic toxicity in experimental groups.

Groups	Mean ± SE			LSD value
	30 days	60 days	90 days	
GI: control	0.036 ± 0.00^Ca^	0.033 ± 0.00^Ca^	0.030 ± 0.00^Ca^	0.005^NS^
GII: 100 mg/kg	2.17 ± 0.03^Bc^	3.59 ± 0.20^Bb^	6.25 ± 0.10^Ba^	0.464^*∗*^
GII: 200 mg/kg	2.86 ± 0.02^Ac^	4.71 ± 0.05^Ab^	12.84 ± 0.74^Aa^	1.50^*∗*^
LSD value	0.083^*∗*^	0.426^*∗*^	1.51^*∗*^	—

Results are expressed as mean (ppm) ± standard deviation. Means with the same letter are not significantly different. Means with different big letters in the same column and small letters in the same row are significantly different (^*∗*^*p* ≤ 0.05).

## Data Availability

All our data will be available either in the scientific library of the Faculty of Sciences of Sfax (Tunisia) or in the scientific library of the College of Veterinary Medicine, University AL-Qassim green (Iraq).

## Supplementary Materials

Supplementary Materials Table 1: body weight of animals for chronic toxicity in experimental groups.
