# Supplementary material for: Assessment of Histopathological Alterations and Oxidative Stress in the Liver and Kidney of Male Rats following Exposure to Aluminum Chloride
Source: J Toxicol. 2024 Jul 12;2024:3997463. doi: 10.1155/2024/3997463 (PMC11259504; doi:10.1155/2024/3997463)
Supplement: Supplementary Materials — Table 1: body weight of animals for chronic toxicity in experimental groups. [file 3997463.f1.docx]

**Table.** Body weight of animals for chronic toxicity experimental groups

| **Groups** | |  |  | **Mean (g) ± SE** |  |
| --- | --- | --- | --- | --- | --- |
|  |  | **0 Day** | **30 Days** | **60 Days** | **90 Days** |
| GI: Control | | 252,6 ± 8.7 | 287.8 ± 8.6 | 310.6 ±7.4 | 359.4 ± 6.2 |
| GII: 100mg/Kg | | 256,2 ± 6.4 | 240.2 ± 3.4 | 230.2 ± 4.32 | 215.0 ± 3.8 |
| GII: 200mg/Kg | | 260,8 ± 6.1 | 237.4 ± 6.1 | 228.2 ± 5.76 | 205 ± 3.16 |
|  |  |  |  |  |  |
